# Editor’s introduction

**DOI:** 10.1186/s40854-022-00386-x

**Published:** 2022-09-16

**Authors:** Gang Kou

**Affiliations:** Southwestern University of Finance and Economics, Chengdu, China

The 34th issue of Financial Innovation (FIN), Volume 8, No.4 (2022) presents 15 papers contributed by authors and co-authors from seventeen countries and areas: Australia, Bangladesh, Brazil, China, Czech, Jordan, Korea, Pakistan, Saudi Arabia, Slovak Republic, Spain, Switzerland, Taiwan, Türkiye, UAE, UK and USA.

- Credit Scoring (9 papers)

Regarding to P2P lending, the paper “Default or profit scoring credit systems? Evidence from European and US peer‑to‑peer lending markets” suggests a paradigm shift in modeling credit-risk in the P2P market to prefer profit as opposed to credit-risk scoring models. The paper “A credit scoring model based on the Myers–Briggs type indicator in online peer-to-peer lending” proposes an alternative credit scoring model for P2P lending by extracting typical personality types inferred from the borrowers’ job category. The paper “Regulatory constraint and small business lending: do innovative peer-to-peer lenders have an advantage?” investigates the regulatory advantage conferred on innovative Peer-to-Peer (P2P) lenders, in respect of lending to small businesses.

From the viewpoint of crowdfunding platforms, the paper “A literature review and integrated framework for the determinants of crowdfunding success” conducts a review of extant research on the determinants of crowdfunding success. It provides practical implications for both theory and practice, and directions for future research. The paper “Analysis of crowdfunding platforms for microgrid project investors via a q-rung orthopair fuzzy hybrid decision-making approach” indicates that security is the most important factor in crowdfunding platforms for smart-grid project investors and solar panels should be preliminarily developed to increase the effectiveness of microgrid systems.

The paper “Bank loan information and information asymmetry in the stock market: evidence from China” provides suggestive evidence of a close connection between the credit loan and stock markets. The paper “Measuring the model risk-adjusted performance of machine learning algorithms in credit default prediction” proposes a new framework to quantify model risk-adjustments. The paper “The impact of working capital management on credit rating” finds that the deviation from the optimal working capital adversely affects the credit rating. The paper “Application of a distributed verification in Islamic microfinance institutions: a sustainable model” proposes a theoretical model most suitable for Islamic Microfinance Institutions (MFIs) which enables Islamic MFIs’ to operate together with the existing financial models compliant with Islamic Shariah Law.

- Financial market (4 papers)

The paper “Cloud economy and its relationship with China’s economy—a capital market-based approach” creates a China Cloud Economy Index (CCEI) and find its relationship with the stock market has been getting stronger over time, but the availability of cloud-related policies has weakened this relationship.

The paper “Higher-order dynamic effects of uncertainty risk under thick-tailed stochastic volatility” constructs a model to investigate the impact of uncertainty risk on the transmission channel among macroeconomic sectors and to better analyze the dynamic effect of uncertainty risk on macroeconomics.

The paper “Does communication increase investors’ trading frequency? Evidence from a Chinese social trading platform” suggests that social interaction, especially communication, plays an important role in shaping investing behavior.

The paper “Investor sentiments and stock markets during the COVID-19 pandemic” indicates that proxies for positive and negative investor sentiments seem to be good predictors of stock returns and volatility during the pandemic.

- Corporate Finance (2 papers)

The paper “Raising capital amid economic policy uncertainty: an empirical investigation” suggests that during times of high economic uncertainty, firms raise capital more frequently, choose debt-based instruments, and raise higher volumes of capital.

The paper “Operational and investment efficiency of investment trust companies: Do foreign firms outperform domestic firms?” improves the accuracy of the efficiency measurement and adds a network-based ranking component to rank the top-performing entities.

